# In situ optical sub-wavelength thickness control of porous anodic aluminum oxide

**DOI:** 10.3762/bjnano.15.12

**Published:** 2024-01-31

**Authors:** Aleksandrs Dutovs, Raimonds Popļausks, Oskars Putāns, Vladislavs Perkanuks, Aušrinė Jurkevičiūtė, Tomas Tamulevičius, Uldis Malinovskis, Iryna Olyshevets, Donats Erts, Juris Prikulis

**Affiliations:** 1 Institute of Chemical Physics, University of Latvia, 19 Raina Blvd., Riga LV-1586, Latvia; 2 Institute of Materials Science of Kaunas University of Technology, K. Baršausko St. 59, Kaunas LT-51423, Lithuania; 3 Department of Physics, Faculty of Mathematics and Natural Sciences, Kaunas University of Technology, Studentų St. 50, Kaunas LT-51368, Lithuania; 4 Faculty of Chemistry, University of Latvia, 1 Jelgavas Str., Riga LV-1004, Latvia; 5 Faculty of Physics, Mathematics and Optometry, University of Latvia, 3 Jelgavas Str., Riga LV-1004, Latvia

**Keywords:** electrochemistry, ellipsometry, porous anodic alumina, spectroscopy, thin films

## Abstract

Porous anodic aluminum oxide (PAAO), sometimes referred to as nanoporous anodic alumina, serves as a cost-effective template for nanofabrication in many fields of science and engineering. However, production of ultrathin PAAO membranes with precise thickness in the optical sub-wavelength range remains challenging because of difficulties regarding process control at the initial stage of anodic oxidation. In this study, we demonstrate a technique for consistently manufacturing PAAO with the targeted thickness. An electrochemical cell with an optical window was designed for reflectance spectroscopy of PAAO during anodization. Real-time fitting of spectra to a transfer-matrix model enabled continuous monitoring of the thickness growth of the PAAO layer. Automation software was designed to terminate the anodization process at preset PAAO thickness values. While the concept was illustrated using the widely used method of anodization in a 0.3 M oxalic acid electrolyte with a 40 V potential, it can be readily customized for other protocols. PAAO layers with effective thickness below 300 nm could be produced with a few nanometers accuracy using single-crystal aluminum substrates. The results were confirmed using spectroscopic ellipsometry. The method for controlling the thickness during anodization eliminates the necessity of sample sectioning for electron microscopy and is particularly valuable for the small-scale production of PAAO-based functional optical coatings.

## Introduction

Porous anodic aluminum oxide (PAAO) is a versatile self-organized material with applications in many fields of science and technology, including nanofabrication [1], optical coatings [2], sensing [3–5], and others [6]. Many synthesis protocols have been developed for precise control of the pore structure of PAAO [7], which allow for the creation of nanoscale patterns for various types of templates, including evaporation masks [8–10], molds for nanowire array production using the supercritical fluid method [11], electrochemical deposition [12], atomic layer deposition [13], or traps for colloidal nanoparticle assembly [14]. Several applications, for example, color filtering [15] and optical sensors [4–5], require precise control of PAAO layer thickness in the optical sub-wavelength range. Among other examples, by tuning the thickness of PAAO between 200 and 600 nm, it becomes possible to selectively enhance or suppress photoluminescence (PL) bands originating from defects in zinc oxide nanorods embedded within the PAAO template [13]. Recently, it was demonstrated that the PAAO thickness tuning can increase the signal intensity and refractometric sensitivity of localized surface plasmon resonance (LSPR) sensors constructed using gold nanoparticles, which are deposited on the pore openings of the PAAO [16]. An influence of PAAO thickness variation in the range from 500 nm to 5 μm on biosensor performance using gold-capped PAAO has been reported [17].

Usually, the PAAO thickness is determined by the anodization time and growth rate. Although the thickness accuracy can be improved by slow anodization at low temperatures [18], because of the spontaneous nature of oxide formation at the initial phase of PAAO growth, process timing alone cannot guarantee the desired outcome. Furthermore, the growth rate of the PAAO can be influenced by other factors, including local heating, electrolyte flow [19], arrangement of the electrodes, and crystallographic orientation of the aluminum substrate [20].

In this work, we continuously recorded the reflectance spectra from a PAAO-coated aluminum surface during anodization. In a similar reflective interference spectroscopy (RIfS) setup, the PAAO structure was analyzed using the fast Fourier transform (FFT) method [21]. However, the optimal PAAO thickness for FFT analysis was ≈2.5–5.0 μm, where multiple interference fringes can be observed in the reflectance spectra. Here, the PAAO thickness was calculated in real time by fitting the reflectance spectra to a multilayer model of a water–PAAO–aluminum system using the transfer-matrix method (TMM) [22]. Previously, TMM has been employed only for post-production thickness analysis of PAAO-based stratified systems [13,23]. In the present study, it allowed for continuous in situ monitoring of PAAO layer growth and the termination of the process at a desired PAAO thickness for reliable fabrication of subwavelength optical coatings with thickness below 300 nm.

## Results and Discussion

**Figure 1:**
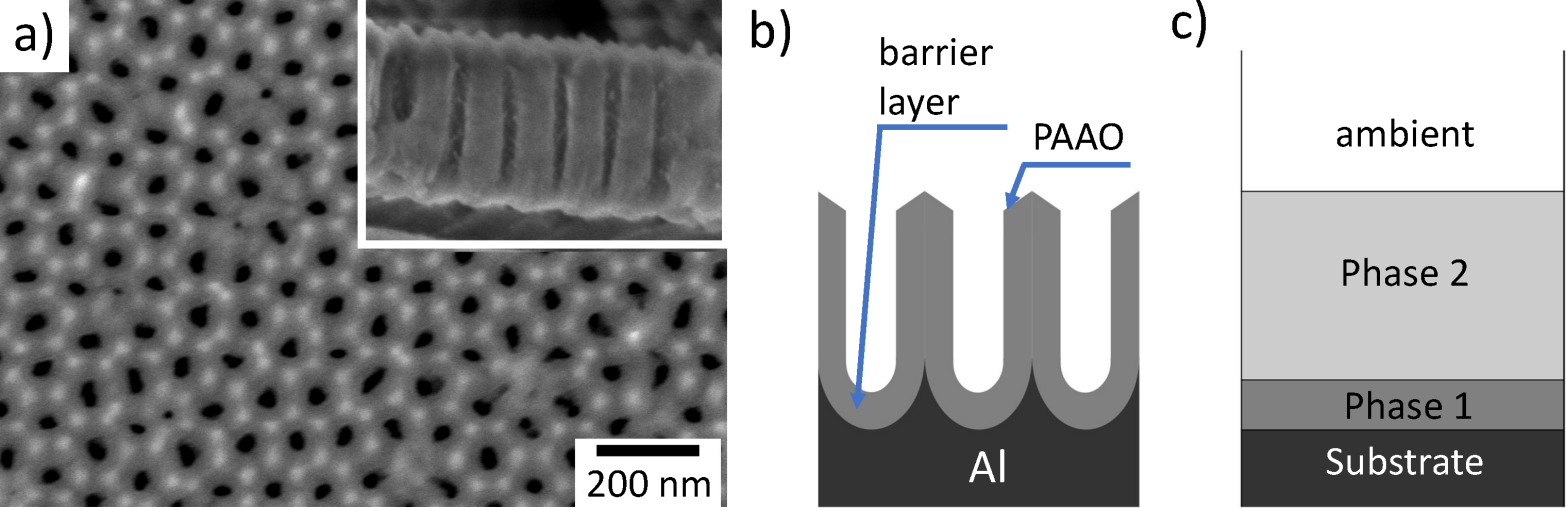
(a) Scanning electron microscopy (SEM) micrograph showing top view and cross section (inset, same scale) of a representative porous anodic aluminum oxide (PAAO) layer, produced through anodization at 40 V using 0.3 M oxalic acid electrolyte. (b) Diagram illustrating the PAAO structure. (c) Schematics of the optical model used in spectroscopic ellipsometry data analysis.

The obtained PAAO layer structure (Figure 1a) with a hexagonal pore arrangement, ≈100 nm center-to-center distance, and ≈30 nm pore diameter corresponded well to the expected results of using anodization in 0.3 M oxalic acid electrolyte and 40 V voltage [24–25]. PAAO is not a homogeneous material; instead, it consists of a porous layer and the barrier layer on top of the Al substrate (Figure 1b). To achieve precise optical characterization, one could employ spectroscopic ellipsometry (SE) with more refined division into sub-layers [26] and consider additional material properties, such as the anisotropy of PAAO [27] and the optical dispersion of the refractive index (RI) of Al_2_O_3_ [28]. However, for consistent thickness determination during anodization using reflectance measurements at normal incidence, it was sufficient to assume a single PAAO layer with the effective RI *n*_eff_. A simplified model consisting of an Al substrate with the complex RI 
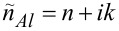
 [29], PAAO with constant *n*_eff_, and water with RI 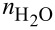 [30] enabled a fast non-linear least squares fit to a TMM model function using the Levenberg–Marquardt (LM) algorithm from the SciPy library [31] for finding the PAAO layer thickness *h*_PAAO_. Typical PAAO growth rates at the steady-state anodization stage using the specific experimental conditions (electrolyte, temperature, voltage, and sample geometry) were found to be less than 1 nm/s. Together with spectrum acquisition, data transfer, and other tasks, it was possible to extract *h*_PAAO_ values at 0.2 s intervals, which resulted in sub-nanometer sampling resolution of *h*_PAAO_. However, it is important to note that *h*_PAAO_ is an integrated (effective) value obtained by collecting spectra from a surface area significantly larger than the microstructure of PAAO, including pores, pore walls, skeleton, and the interstitial rods [32], which have tens of nanometers difference in length.

**Figure 2:**
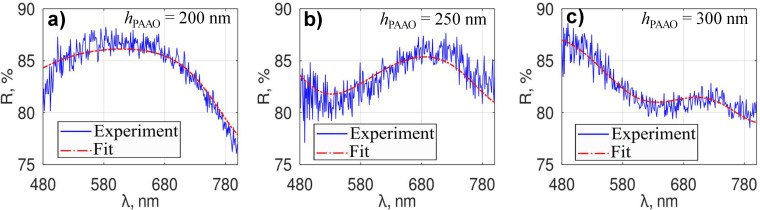
Experimental and simulated (fitted) reflectance spectra from Al–PAAO samples in electrolyte solution after (a) 145 s, (b) 235 s, and (c) 268 s anodization time. The indicated *h*_PAAO_ values are fit results.

Figure 2 shows typical recorded reflectance spectra after different anodization times together with simulated spectra using the TMM model function. The spectral shape is determined by the partial transmission at the electrolyte–PAAO interface and multiple reflections within the PAAO layer, which results in interference minima and maxima at different wavelengths. The usable wavelength range was limited primarily by the low intensity of the incandescent light source in the violet part of the spectrum and the sensitivity of the detector in the infrared. Other materials (fiber, glass, electrolyte, and coolant) in the optical path can further attenuate the signal at short and long wavelengths.

**Figure 3:**
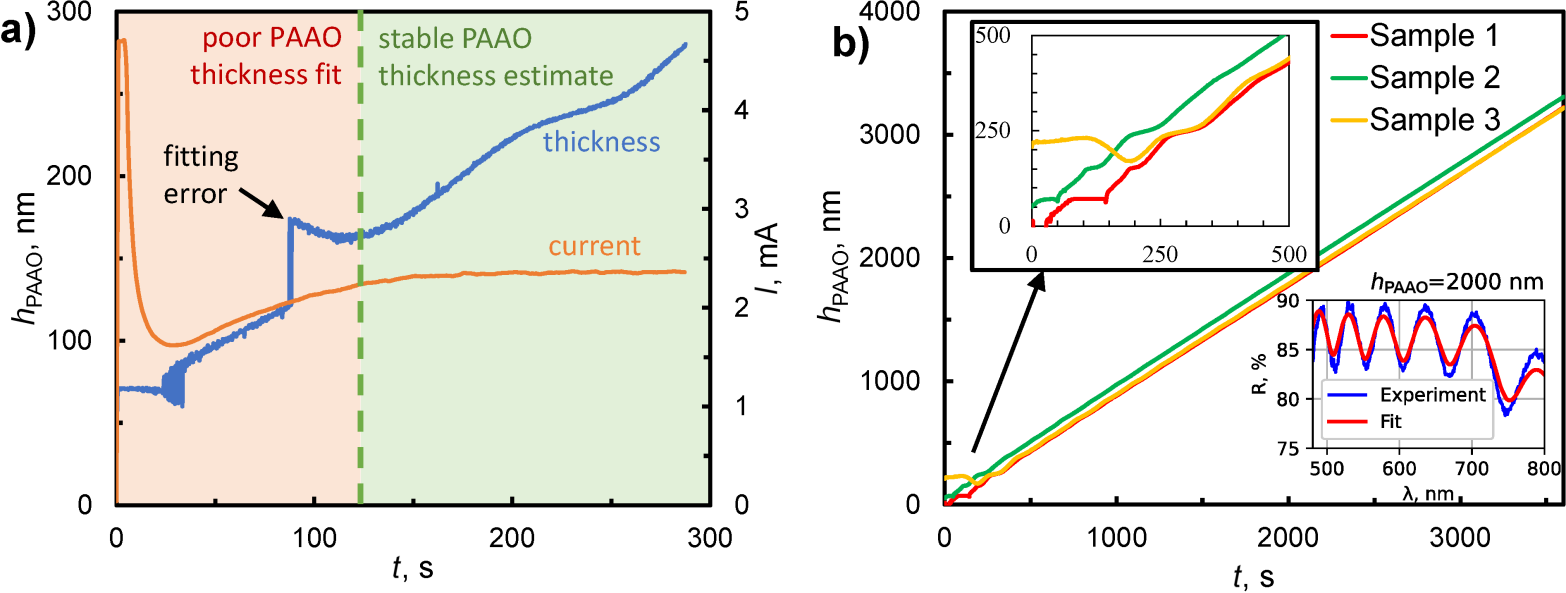
(a) Example of a real-time measurement of PAAO layer thickness and current through a 1 cm^2^ anode during the second anodization. (b) Measured PAAO layer thickness during the first anodization. The inset (lower right) shows representative measured and fitted spectra for a relatively thick (2 μm) PAAO layer during anodization.

For very thin PAAO layers (*h*_PAAO_ *<* 200 nm), the reflectance spectra did not have significant interferometric features (i.e., Fabry–Pérot-like fringes) in the usable wavelength range of the system. Furthermore, as will be discussed later, at the initial stage of anodization, the alumina layer may not be correctly represented by a single-layer effective RI. This typically resulted in fitting errors during the first 1–2 min of anodization (*h*_PAAO_ *<* 200 nm) as shown in Figure 3a. Moreover, the LM algorithm is sensitive to the initial guess value of the fit parameters and may converge to a bad local minimum [33] resulting in a wrong thickness value.

In order to improve the thickness estimate, the initial guess value *h*_guess_ for the LM algorithm was calculated using the formula *h*_guess_ = 0.81*t* + 71.6 (nm), where *t* is the anodization time in seconds. This relation was obtained by extrapolating data from successful fits of prerecorded spectra from a number of samples during steady-state growth of PAAO and needs to be adjusted for different sets of anodization conditions (e.g., electrolyte and voltage). At a thickness near 200 nm the LM algorithm captured the correct *h*_PAAO_ values. The onset of the stable thickness capture mode coincided with the beginning of the plateau observed in the anodization current kinetics curve (Figure 3a), which corresponds to the steady-state growth of PAAO [7]. While the primary focus of this study is on producing sub-wavelength PAAO layers, Figure 3b illustrates that the thickness estimate using a fit to the TMM model can effectively monitor PAAO thicknesses well exceeding 1 μm. The linear increase of PAAO thickness with time during steady-state growth of PAAO using a constant anodization voltage is in agreement with other works [21]. However, for short anodization times (thin PAAO films, *h*_PAAO_ *<* 300 nm) the relationship may no longer be linear, and the current kinetic curves may exhibit variability among different samples [10].

**Figure 4:**
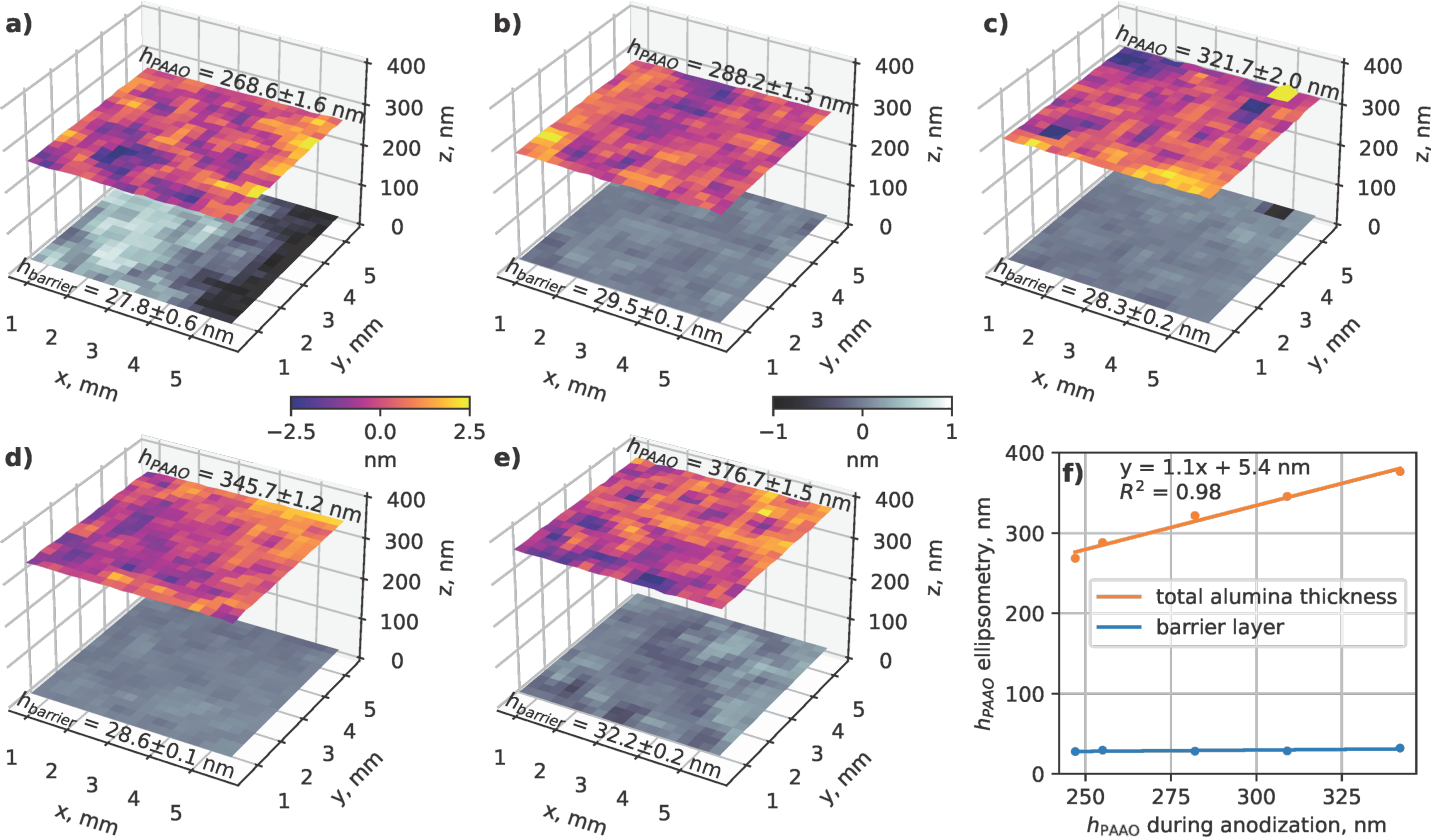
Thickness maps of total PAAO layer and barrier layer obtained using spectroscopic ellipsometry from single-crystal aluminum samples after anodization times of (a) 196 s, (b) 217 s, (c) 248 s, (d) 270 s, and (e) 297 s. The color scale bars represent deviations from the mean value in nanometers. (f) Comparison of PAAO thickness estimates obtained during anodization and post-production using SE.

To confirm the accuracy of the thickness measurements and assess the consistency of the PAAO layer, several samples were mapped via SE. Instead of a single-layer alumina with effective RI *n*_eff_, the model for SE measurements (Figure 1c) consisted of a barrier layer (phase 1) and a porous alumina layer (phase 2). As can be seen in Figure 4a–e, the thickness variation of the total alumina film was within 1–2 nm standard deviation on all samples. There was a linear relation between thickness measurement during anodization and post-production analysis using SE, with a slope coefficient of 1.1 and a constant offset of 5.4 nm (Figure 4f). The deviation from the ideal 1:1 relation can be explained by the differences in effective RI values and the inclusion of the barrier layer in the SE model. The obtained barrier layer thickness was constant (approximately 30 nm) for all samples (Figure 4). This is similar to values of 30–40 nm reported in other studies using the same 0.3 M oxalic acid electrolyte [34–35] after prolonged anodization times. However, during the first 2–3 min the barrier layer thickness can vary substantially [36], potentially attributed to changes of current density [35] and local temperature [19]. Furthermore, the barrier layer is not flat and contains some amount of aluminum that alters the effective refractive index [26].

Several different RI values for 40 V/0.3 M oxalic acid-type PAAO in air have been reported in the literature, for example, 1.43 at 600 nm wavelength [23], 1.55 for the visible and NIR spectral range [37], or calculated using the simple formula 1.76 − 0.76*f* for the visible band [4], where *f* is the porosity. In the present work, a RI value of 1.40 ± 0.01 at 600 nm was obtained for the porous layer in air using SE fitting, while the corresponding value for the barrier layer was 1.768 according to the SE software database for aluminum oxide. During anodization, however, the pores are filled with electrolyte and reaction products. Therefore the value *n*_eff_ = 1.65 used for in situ thickness control was higher than that of dry PAAO and lower than that of pure Al_2_O_3_. It resulted in satisfactory fits of the TMM model function to the recorded reflectance spectra (Figure 2).

Above considerations set the lower limit of applicability of presented thickness monitoring through fitting to a single layer TMM model. Below approximately 200 nm thickness, the alumina layer contains a significant volume of material with uncertain composition, which would require a refined model with multiple phases and make real-time fitting difficult. However, for a thickness of approximately 250–300 nm and above, fitting to a single PAAO layer and an effective RI is sufficient for reliable in situ thickness monitoring. For improved accuracy, the system can be calibrated using post-production analysis (Figure 4f). The potential extension of the interferometric thickness measurement method to thinner PAAO layers could involve the utilization of a shorter-wavelength light source and UV-compatible optical components. In such a scenario, considerations should be made for the absorption and photoluminescence characteristics of PAAO [38].

In order to achieve nanometer-scale thickness uniformity of the PAAO layers (Figure 4), it was necessary to use single-crystal aluminum substrates as starting material. In previous studies it was shown that anodization of polycrystalline aluminum can result in tens of nanometers PAAO thickness variation due to different anodization reaction rates on surfaces with different crystallographic orientation [20,23]. Additionally, it is known, that anodization of aluminum substrates with (100) surface orientation result in better pore ordering in comparison with other crystallographic planes [20,39]. It should be mentioned that the high homogeneity in the effective PAAO thickness (Figure 4) is attainable within the central region of the sample. However, notable variations may occur near sample edges and corners.

Regarding the applicability of the presented method to other anodization protocols and film growth in a broader context, an important assumption for using the TM model is that the pore diameters (or other inhomogeneities in general) are much smaller than the wavelength of light and the film can be characterized by an effective medium refractive index. For instance, PAAO produced using phosphoric acid electrolyte at 120 V [40] can have 193 nm mean spacing for hexagonal pore arrangement and 14.4% pore volume. In comparison to oxalic or sulfuric alumina films [40] (with significantly smaller pore spacing and absolute volume) the phosphoric alumina, has a much higher optical density, which is attributed to scattering by pores. Furthermore, the pore uniformity and ordering can contribute to optical scattering.

## Conclusion

We have developed an interferometric system to enable real-time monitoring and control of the anodization process, ensuring consistent production of PAAO films with thicknesses of approximately 250–300 nm and above. The method relies on fitting the measured reflectance spectra to a transfer-matrix model that features a single alumina layer with a constant effective refractive index. The thickness values obtained in situ during anodization were confirmed using post-production spectroscopic ellipsometry, showing 1–2 nm variation (standard deviation) within each sample. The process is inherently non-invasive and eliminates the need for slicing the sample to measure thickness, as one might do with electron microscopy, for instance. This makes it particularly suitable for quality control in the small-scale production of thin PAAO membranes for optical applications and other uses, where precise thickness is of importance.

## Experimental

**Figure 5:**
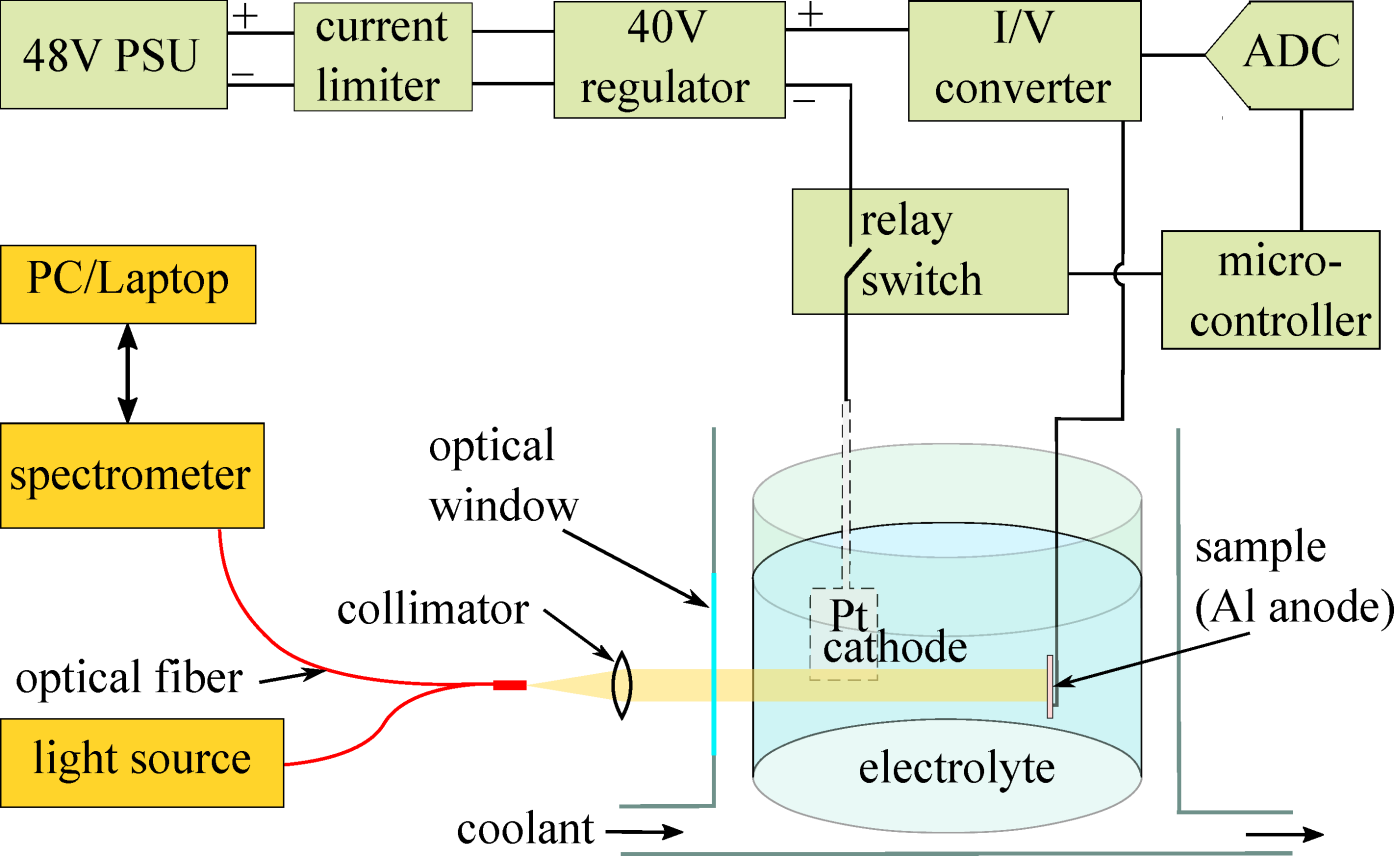
Schematic diagram of the anodizing apparatus.

A dedicated setup (Figure 5) was built and optimized for anodization of 10 mm × 10 mm aluminum samples at constant 40 V potential. Platinum cathode and single crystal Al(100) (MTI Corp. mcALa101010) anode were immersed in 0.3 M oxalic acid electrolyte inside a multiwalled container with a transparent optical window. The container was placed on a magnetic stirrer and cooled to 5 °C. The reflectance spectra were collected using a bifurcated optical fiber (LIGHTGUIDE LGO.INT-06.2020) with one branch attached to a stabilized tungsten-halogen light source (THORLABS SLS201L/M ) and the other to a miniature spectrometer (OCEAN OPTICS USB4000). The common optical fiber port was collimated to a ≈2 mm diameter beam and directed normally to the sample surface. The reflection spectrum from a polished Al sample inside the anodization container was used as a reference for normalization of the reflectance spectra. The reflectance spectra were collected periodically at 200 ms intervals using Ocean Optics SpectraSuite software and stored locally on a PC/laptop. Every new spectrum was used to fit a TMM model function to extract the total PAAO thickness *h*_PAAO_. The model function was constructed using propagation and transmission matrices [22,41] with a constant *n*_eff_ = 1.65 for PAAO layer and tabulated RI values for Al [29] and water [30]. The model function calculated the reflectance spectra with two fitting parameters, that is, PAAO thickness *h*_PAAO_ and a constant multiplier for intensity correction of the reference spectrum. Fitting was done using the Levenberg–Marquardt (LM) algorithm as described in the “Results and Discussion” section. The software source code is available on GitHub [42].

The anodization electronics (marked green in Figure 5) was galvanically isolated from the rest of the system and controlled using the wireless interface of the microcontroller (ESP8266, Espressif Systems). Voltage from a generic telecom 48 V power supply in series with a 150 Ω current-limiting resistor was stabilized to 40 V using a LM317HV-MIL (Texas Instruments) voltage regulator, which is rated for 1.25 to 57 V output voltage range. Thus, with minimal component value changes the apparatus can be customized for other anodization voltages. The current flow through the Pt cathode could be established or interrupted automatically through a relay switch triggered by the thickness measurement software upon reaching the predefined threshold value. The anode current was monitored using a current-to-voltage (*I*/*U*) converter and digitized using an analog-to-digital converter (ADS1115, Texas Instruments). The electronics design files (KiCAD) and microcontroller software source code are accessible on GitHub [43].

The two-step anodization procedure in 0.3 M oxalic acid electrolyte at 40 V potential has been described in literature many times [4,8,10,15–17,20]. In the present work, the single-crystal Al(100) substrates were used as received without electropolishing. The time for the first anodization was 60 min. The second anodization was automatically terminated by the thickness controlling software.

Several weeks after production, the samples were investigated using a rotating-compensator, variable-angle spectroscopic ellipsometer GES5-E (Semilab). Ellipsometric parameters were registered in the wavelength range of 250–950 nm. The angle of incidence was from 55° to 75° in steps of 5°. The measurements covered the central 5 × 5 mm^2^ part of the sample surface, in a grid of 20 × 20 with 254 μm distance between each position. The spot size for SE mapping was 365 μm × 470 μm at 75° angle of incidence. The fitting of the optical model to the experimental data was done in Spectroscopic Ellipsometry Analyzer software (SEA, v1.3.8, Semilab). The optical model is depicted in Figure 1c. The substrate is aluminum, phase 1 represents the barrier layer and is aluminum oxide, phase 2 represents PAAO and is a mixture of air and aluminum oxide, and ambient is air. The optical properties of all materials were available in the built-in *n*,*k* database. The mixture was described as Bruggeman effective medium approximation [44]. The experimental spectra were smoothed by built-in spline smoothing tool prior to fitting. Variables changed during fitting were thicknesses of phases 1 and 2, and volume concentrations of materials in phase 2. A simulated annealing fitting algorithm was employed.

The PAAO structure (Figure 1a) was confirmed using field-emission scanning electron microscopy (FE-SEM-4800, Hitachi, Tokyo, Japan). The relationship between thickness measurements using SEM and optical interferometry has been established in other works, for example [13].

## Funding

The work was performed within the Latvian Council of Science fundamental and applied research project LZP-2020/1-0200 “Nanostructured multilayer hybrid coatings for interferometric and optoelectronic sensors” and European Union’s Horizon 2020 Research and Innovation Program under TRANSLATE project (Grant agreement: 964251). A. J. acknowledges support from European Regional Development Fund for postdoctoral project ”Patterned hybrid multilayer films for optical sensors” (no. 1.1.1.2/VIAA/4/20/615).
